# Supplementary material for: Pulsed electrosynthesis orthogonally optimizes C‒N coupling and hydrogenation for amine production with a molecular catalyst
Source: Nat Commun. 2026 May 4;17:4027. doi: 10.1038/s41467-026-72678-0 (PMC13139404; doi:10.1038/s41467-026-72678-0)
Supplement: Supplementary file 1 — Supplementary Information [file 41467_2026_72678_MOESM1_ESM.pdf]

Pulsed Electrosynthesis Orthogonally Optimizes C–N Coupling and Hydrogenation for Amine Production with a Molecular Catalyst

Shuai Yan[1], Yang Wang[1], Shuai Chen[1], Jost Heise[1], Morgan McKee[1], Chen Gao[1], Xiaodong Li[2,3*], Nikolay Kornienko[1*]

[1]Institute of Inorganic Chemistry, University of Bonn, Gerhard-Domagk-Str. 1, 53121 Bonn, Germany

[2]Max Planck Institute of Microstructure Physics, Weinberg 2, Halle 06120, Germany

[3]Key Laboratory of Precision and Intelligent Chemistry, University of Science and Technology of China, Hefei, Anhui 230026, China

*Corresponding Authors

*Email: Xiaodong.li@tu-dresden.de, nkornien@uni-bonn.de

Table of Contents:

Supplementary Note 1

Supplementary Figures 1–39

Supplementary Tables 1–5

Supplementary References 1−18

Supplementary Note 1. Techno-Economic Assessment

A hypothetical chemical plant was designed to estimate the production cost of methylamine using CO_2 and KNO_3 as chemical feedstocks. The plant operates with a fixed input power (P_{in}) of 10 MW. The total production cost per tonne of product was divided into four main components: feedstock cost, electricity cost, plant operational cost, and capital cost associated with plant construction. It should be noted that unit conversions are not explicitly shown in the following equations.

Feedstock cost

To determine the feedstock cost, the required amounts of input chemicals (m_r) were calculated based on the molar masses of the reactants (M_r) and the product (M_p). For example, the mass of CO_2 required to produce one tonne of methylamine is calculated as:

$$m_{CO_2} = \frac{1\ tonne}{M_p} * M_{CO_2} \tag{1}$$

The total expenditure on feedstock for producing one tonne of methylamine was obtained by summing the cost of all reactants. Market prices for the input chemicals were sourced from literature, manufacturers, or commercial databases. For the cost estimation, the price of CO_2 is set at US$70 per tonne, assuming conventional sourcing.[1,2] The price of KNO_3 used as the nitrate source, is set at US$700 per tonne.[3] Thus, the feedstock cost is given by the product of each reactant mass and its corresponding market price ($\mathrm{Cost_r}$):

$$\text{Feed Cost} = \sum m_r * Cost_r \tag{2}$$

Electricity cost

To estimate the electricity cost associated with the electrochemical reactions, the total charge required to produce one tonne of product (Q) should considered:

$$Q = 1\ tonne * z * F/(M_p\ * FE * EE) \tag{3}$$

In this equation, EE represents energy efficiency. The consumed energy (E) depends on the charge Q and the cell voltage (U_{cell}):

$$E = Q * U_{cell} \tag{4}$$

To simulate the impact of pulsed electrolysis, a square-wave potential was designed with time intervals t_1 and t_2 and corresponding applied potentials U_1 and U_2. The applied potentials were determined with respect to the oxygen evolution reaction (OER) as the counter-reaction. The potential for OER was assumed to be 1.8 V vs. RHE. Since the energy consumption was previously calculated using a constant potential, the alternating energy supply during pulsed electrolysis needs to be approximated by an average potential, calculated as:

$$U_{cell} = \frac{|U_{OER} - U_1| * t_1 + |U_{OER} - U_2| * t_2}{t_1 + t_2} \tag{5}$$

The electricity expenditure per tonne product is then calculated by multiplying the energy consumption by the electricity price:

$$Electricity\ cost = E * electricity\ price \tag{6}$$

Capital Cost

A significant portion of the product cost arises from capital expenditures (CapEx), which include investment in necessary equipment. Since detailed data on large-scale C–N bond electrolyzers are scarce, cost information from similar electrochemical systems, such as CO_2 reduction reactors and water electrolyzers, was utilized. The electrolyzer is the central equipment in an electrocatalytic plant. To estimate its cost, the required catalytic surface area and unit cost per area must be determined. Here, the electrolyzer cost was approximated by analogy to a proton exchange membrane water electrolyzer, which uses membranes loaded with iridium and platinum. The estimated cost per square meter of catalytic surface was set at approximately US$3000, covering both the membrane and additional material like gasket.[4] It is worth noting that advancements in electrolyzer design are expected to significantly lower capital costs in the coming decades.[2]

The techno-economic analysis fixed the input power as a key parameter to calculate the electrolyzer's required active area (A), based on cell voltage and measured current density (j):

$$A = P_{in}/(U_{cell} * j) \tag{7}$$

The total electrolyzer cost ($Cost_{electrolyzer}$) can thus be estimated by multiplying this area by the unit cost:

$$Cost_{electrolyzer} = A * \text{US\$}3000/m^2 \tag{8}$$

Additional plant expenses come from separation, pulsed electrolysis equipment (pulse generators), and installation. Given the complexity and variability of these components, their costs were approximated as percentages of the electrolyzer cost to simplify calculations. An installation factor (α_1) of 20% was applied based on relevant studies.[5] The pulse generator cost factor (α_2) is included only for pulsed electrolysis and can be omitted in the case of static electrolysis. Separation costs (α_3) were estimated as 10% of the electrolyzer cost. The total capital cost is expressed as:

$$Cost_{plant} = Cost_{electrolyzer} * (1 + \alpha_1 + \alpha_2 + \alpha_3) \tag{9}$$

As it is uncommon for companies to fund chemical plants entirely through equity capital, a capital recovery factor (CRF) is introduced to estimate the annualized investment cost. The CRF reflects the fixed annual payment needed to recover the initial capital expenditure over a given project lifetime at a specified interest rate.[6] Assuming a plant lifetime of n = 20 years and an interest rate i = 8%, the CRF is calculated as:

$$CRF = \frac{i(1+i)^n}{(1+i)^n - 1} \tag{10}$$

The annualized capital cost is obtained by multiplying the total plant investment by the CRF. Dividing this value by the annual production output yields the capital cost per tonne of product:

$$CapEx = Cost_{plant} * CRF/m_p \tag{11}$$

m_p represents the total annual production of the target product from the plant, calculated using the following equation:

$$m_p = j * FE * A * M_p * t/(F * z) \tag{12}$$

where t corresponds to one year.

Operational Cost

Operational expenditures refer to the recurring costs incurred during plant operation, excluding initial capital investments. These mainly include membrane replacement, product separation, and personnel-related expenses. Separation costs ($Cost_{sep}$), which depend on the chemical properties of the target product and purification methods, were approximated as 30% of the total electricity cost for simplicity. Personnel and maintenance costs—covering operators, technical support, and routine upkeep—were estimated at US$800,000 annually.

Membrane degradation necessitates periodic replacement, representing a significant portion of operational expenses. Assuming partial recycling and a replacement cost of $1000 per square meter per cycle, the membrane lifespan (t_{life}) was set to 1.5 years.[7] The annualized membrane replacement cost (MR) is calculated as:

$$MR = A * \text{US\$}1000/t_{life} \tag{13}$$

The total operating cost per tonne of product is expressed as:

$$Operating\ Cost = \frac{Cost_{sep} + salaries}{m_p} + MR \tag{14}$$

Finally, the overall production cost per tonne is obtained by summing feedstock, electricity, capital recovery, and operational expenses. Although revenue from purified byproducts could partially offset costs and improve economic feasibility, such potential income was excluded due to considerable uncertainty. A summary of all parameters used in this analysis is provided in **Supplementary Table 5**.

Supplementary Figures

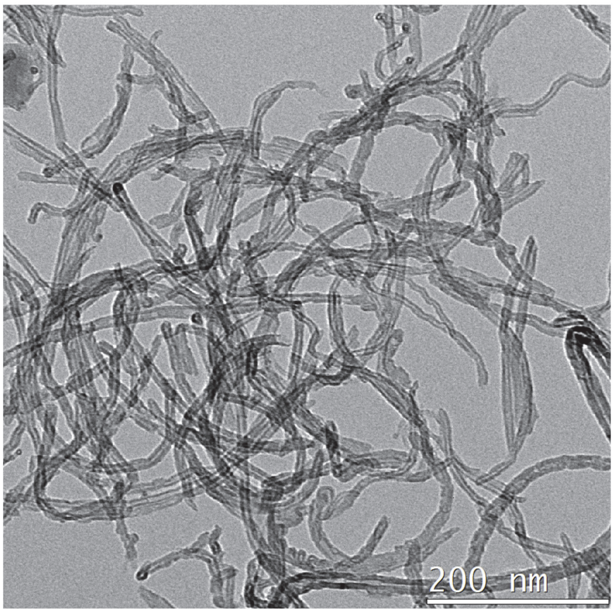

Supplementary Fig. 1 | TEM images of CoPc/CNTs.

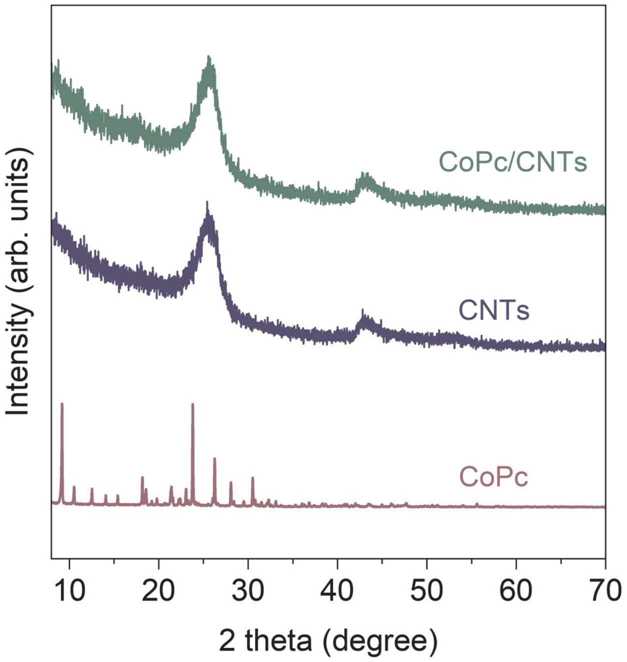

Supplementary Fig. 2 | XRD patterns of CoPc/CNTs, CoPc and CNTs. Source data for Supplementary Figure 2 are provided as a Source Data file.

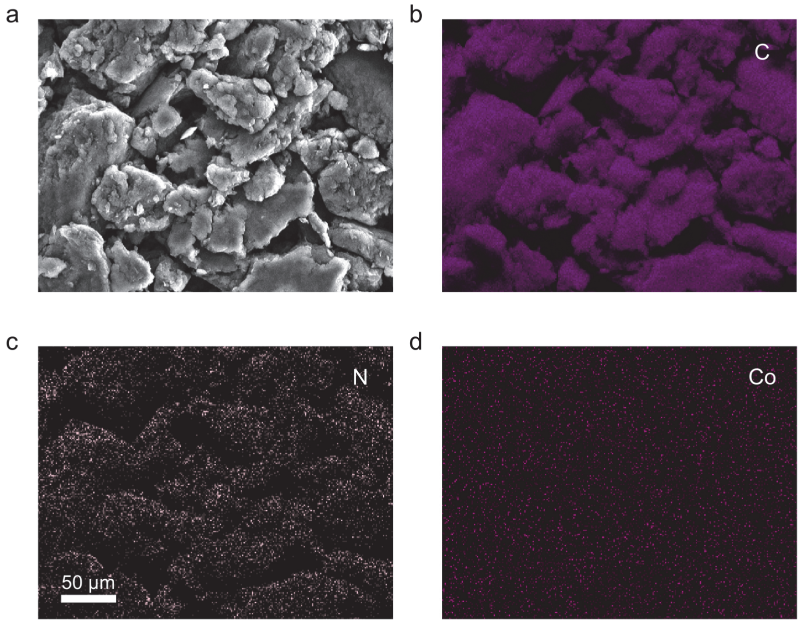

Supplementary Fig. 3 | Morphological characterization of the catalyst. (a) SEM image of the CoPc/CNTs powder. (b–d) Corresponding EDS mapping showing the distribution of C, N, and Co.

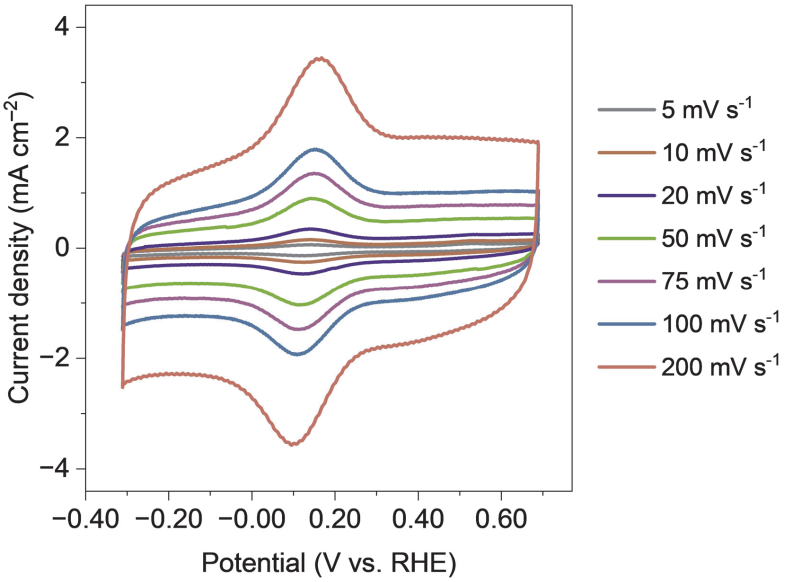

Supplementary Fig. 4 | The linear dependence of peak current density on scan rate (ranging from 5 to 200 mV/s) using CoPc/CNTs catalyst (without iR-correction). Source data for Supplementary Figure 4 are provided as a Source Data file.

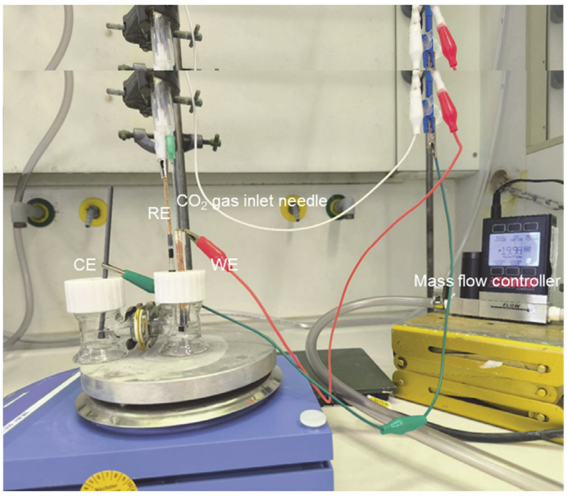

Supplementary Fig. 5 | Photograph of the homemade H-cell used for electrochemical C–N coupling, consisting of two compartments separated by an anion exchange membrane (Fumsep FAA-3-PK-75, 70–80 μm thick). CO_2 was continuously supplied to the cathodic compartment at a flow rate of 20 mL min^{-1} by an ALICAT digital mass flowmeter.

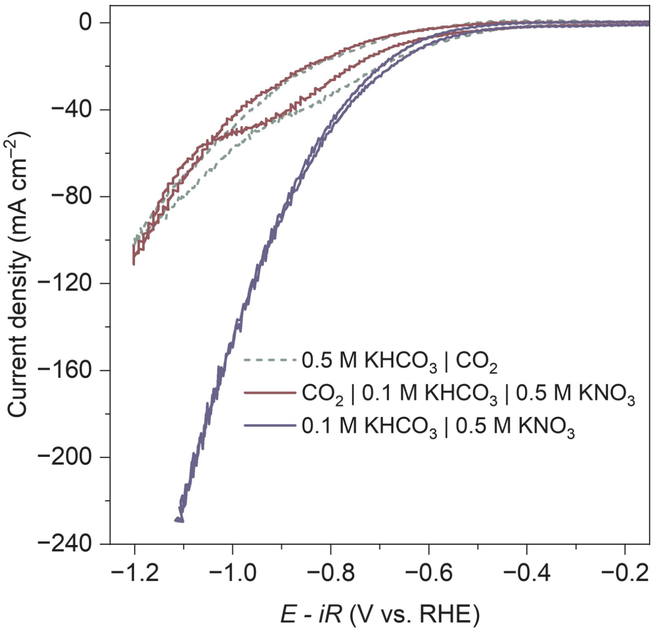

Supplementary Fig. 6 | CVs of the CoPc/CNTs showing CO_2 introduction supressed NO_3^- reduction and H_2 evolution, thus favoring C–N coupling. Source data for Supplementary Figure 6 are provided as a Source Data file.

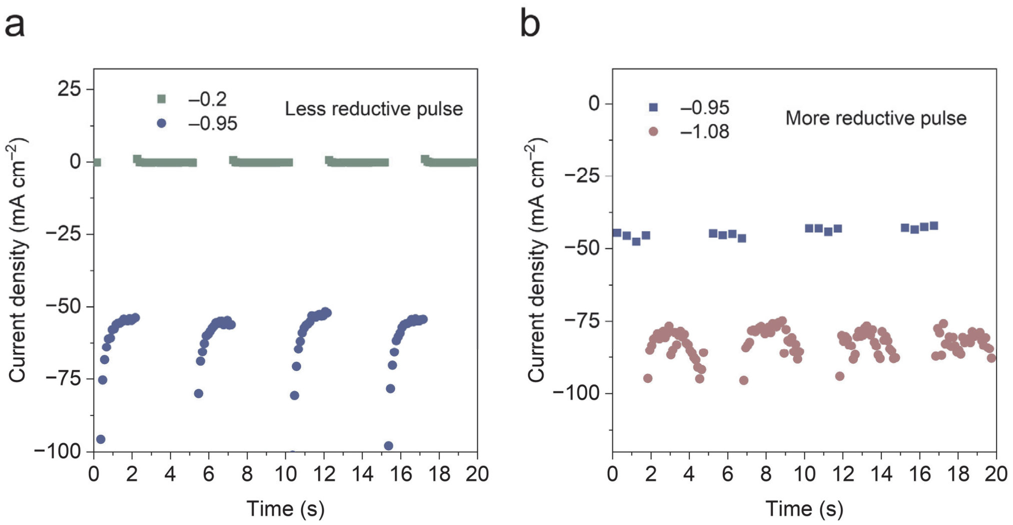

Supplementary Fig. 7 | Chronoamperometric profile under less reductive pulse (a) and more reductive pulse (b). Source data for Supplementary Figure 7 are provided as a Source Data file.

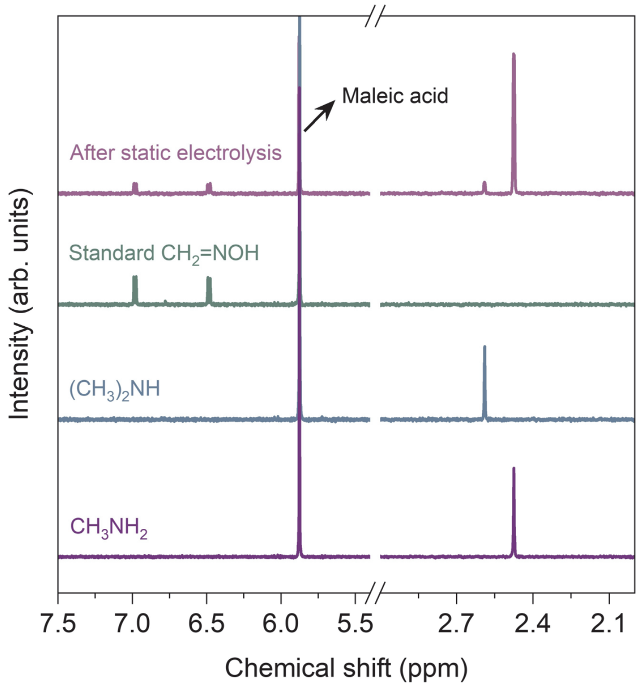

Supplementary Fig. 8 | ^{1}H-NMR spectrum of standard methylamine, dimethylamine and formaldoxime in 0.1 M $KHCO_3$ and 0.5 M KNO_3, and electrolytes after static reaction. Maleic acid was used as the internal standard. Source data for Supplementary Figure 8 are provided as a Source Data file.

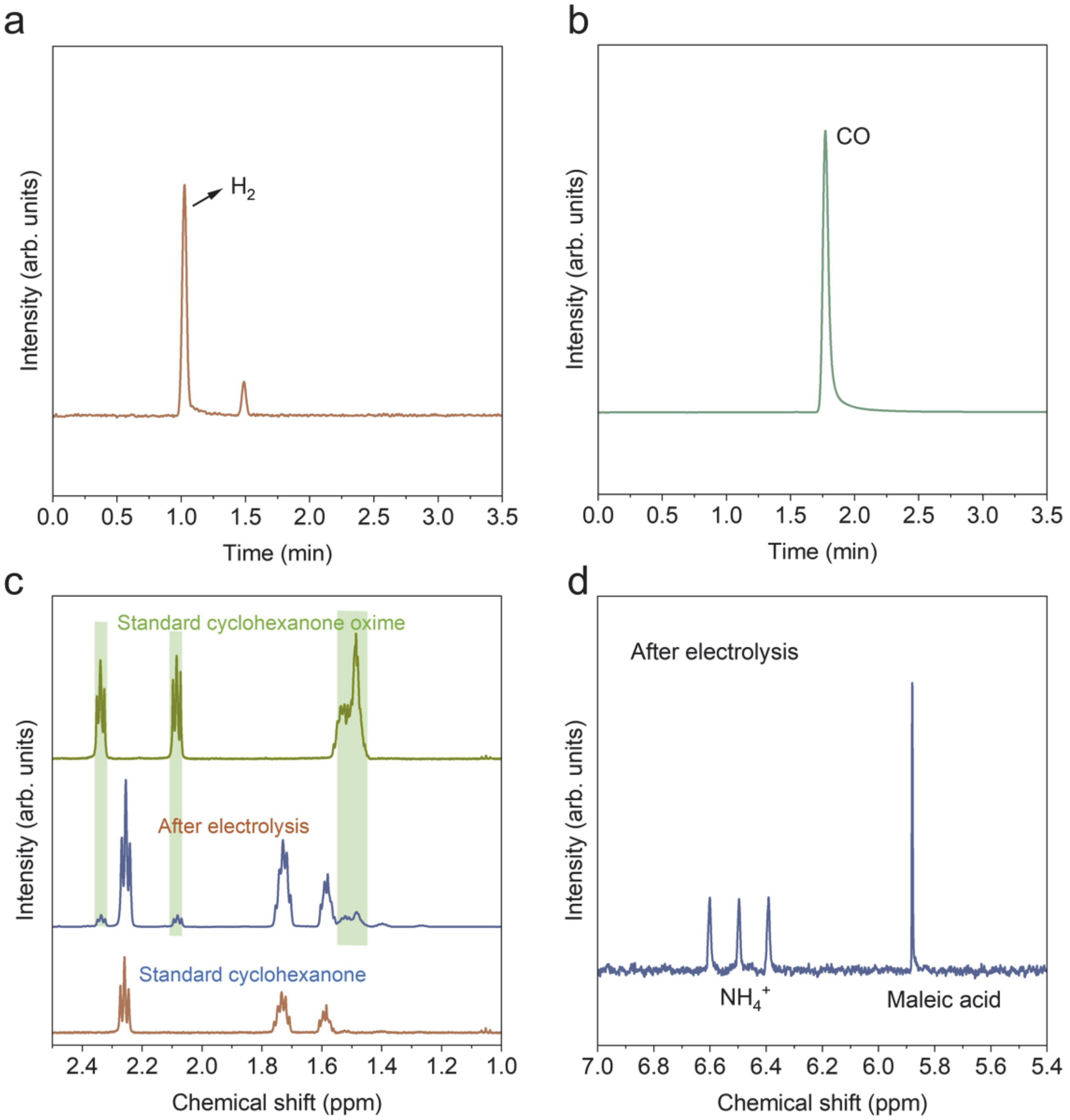

Supplementary Fig. 9 | Gas chromatography results of H_2 (a); and CO (b). ^{1}H-NMR spectra of standard cyclohexanone, cyclohexanone oxime, and electrolytes after electrolysis (c). ^{1}H-NMR spectra of NH_4^+ in electrolytes after electrolysis (d). Source data for Supplementary Figure 9 are provided as a Source Data file.

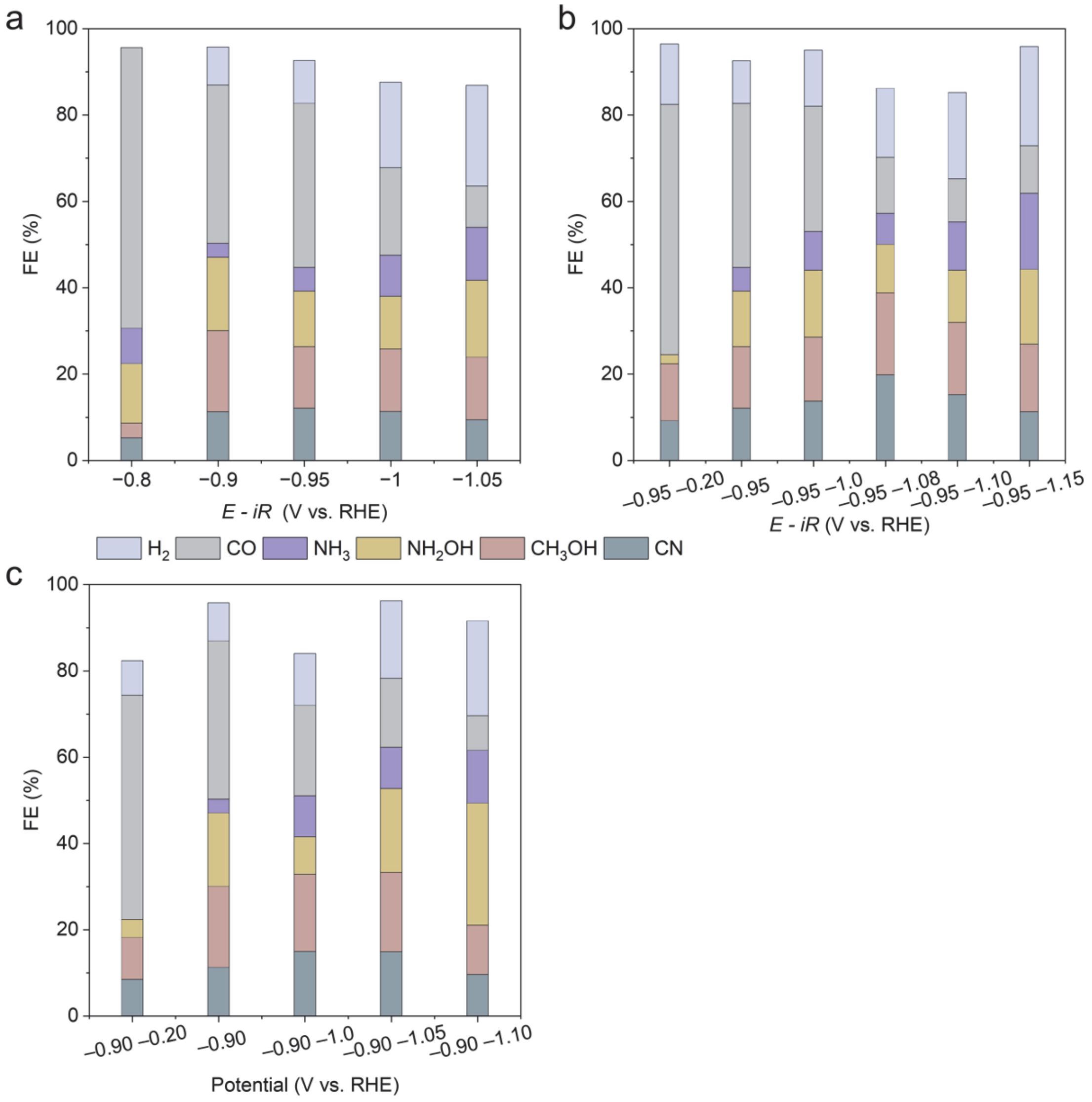

Supplementary Fig. 10 | Product distribution under static potentials (a) and pulsed potentials (b, c). Source data for Supplementary Figure 10 are provided as a Source Data file.

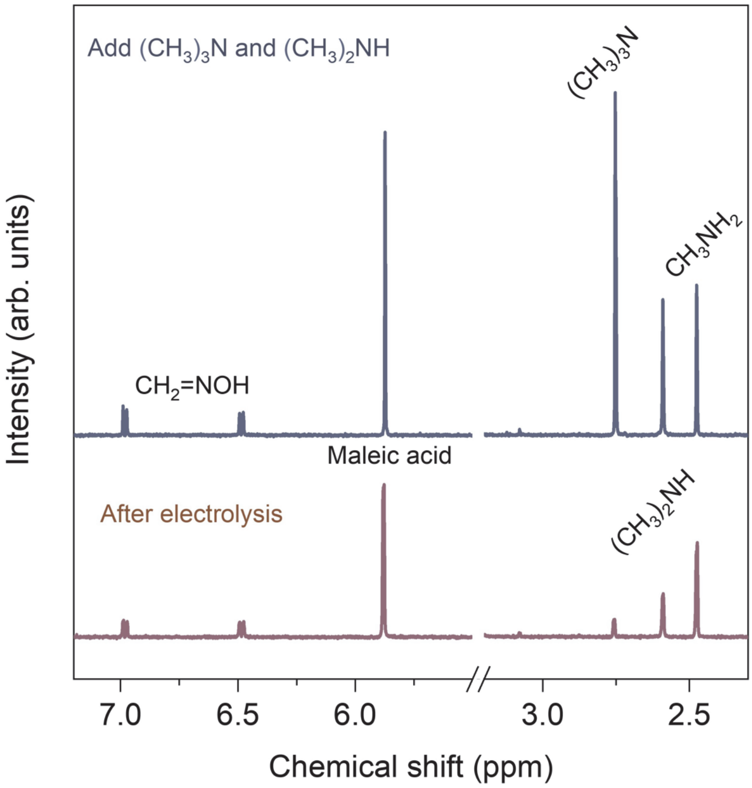

Supplementary Fig. 11 | ^{1}H-NMR spectra for product identification. ^{1}H-NMR spectra of the electrolyte collected after pulsed electrolysis (bottom) and the same electrolyte spiked with a small amount of dimethylamine and trimethylamine standards (top). The selective enhancement of the original peak intensities without the appearance of any new signals unambiguously confirms the assignment of dimethylamine and trimethylamine standards as the electrolysis products. Source data for Supplementary Figure 11 are provided as a Source Data file.

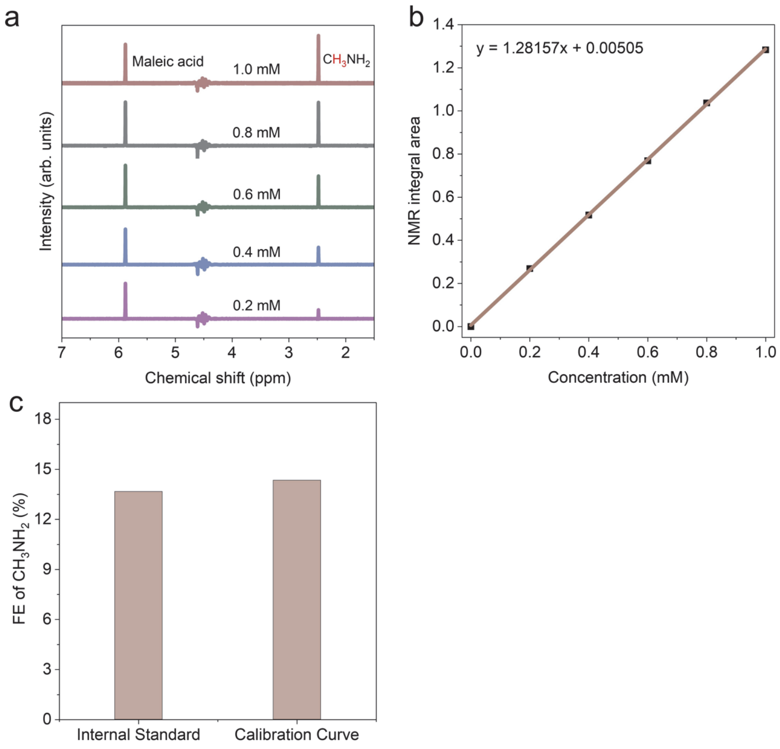

Supplementary Fig. 12 | (a) ^{1}H NMR spectra of methylamine solutions at different concentrations. The solutions were prepared by first making a 1 L solution of methylamine in 0.1 M $KHCO_3$ and 0.5 M KNO_3, followed by serial dilution with the same electrolyte to the desired concentrations. (b) Calibration curve obtained from peak areas normalized to maleic acid (internal standard, area = 1). (c) Comparison of Faradaic efficiencies determined using the internal standard (IS) method and the calibration curve method. Note: All solutions were prepared and stored at 4 °C to minimize volatilization. Source data for Supplementary Figure 12 are provided as a Source Data file.

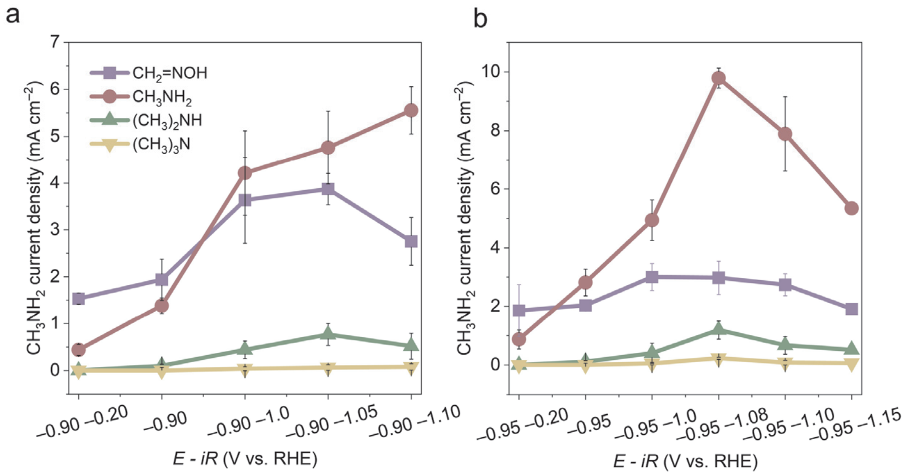

Supplementary Fig. 13 | Partial current densities of C-N products for static and pulsed potentials using –0.90 V (a) and –0.95 V (b) as the reference point, respectively. Source data for Supplementary Figure 13 are provided as a Source Data file.

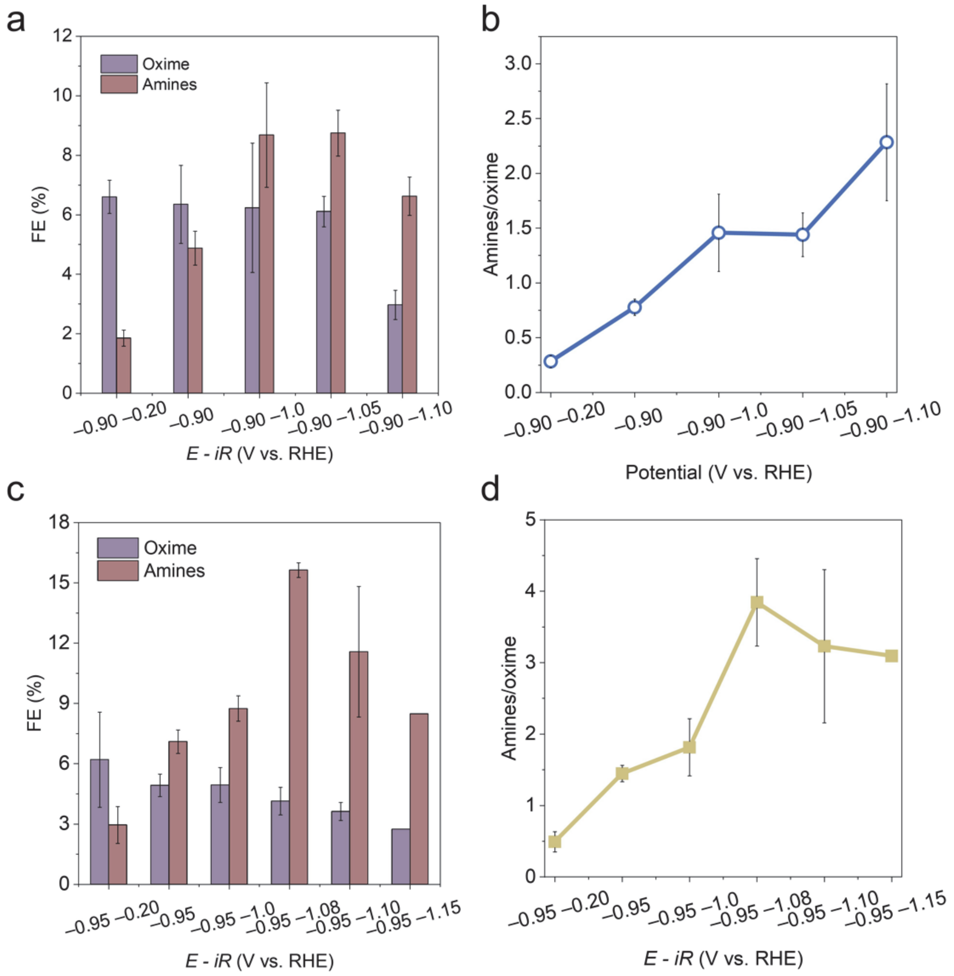

Supplementary Fig. 14 | More reductive pulse promoted hydrogenation from formaldoxime to amines while less reductive pulse shows slow hydrogenation starting from –0.90 V (a,b) and –0.95 V (c,d). Source data for Supplementary Figure 14 are provided as a Source Data file.

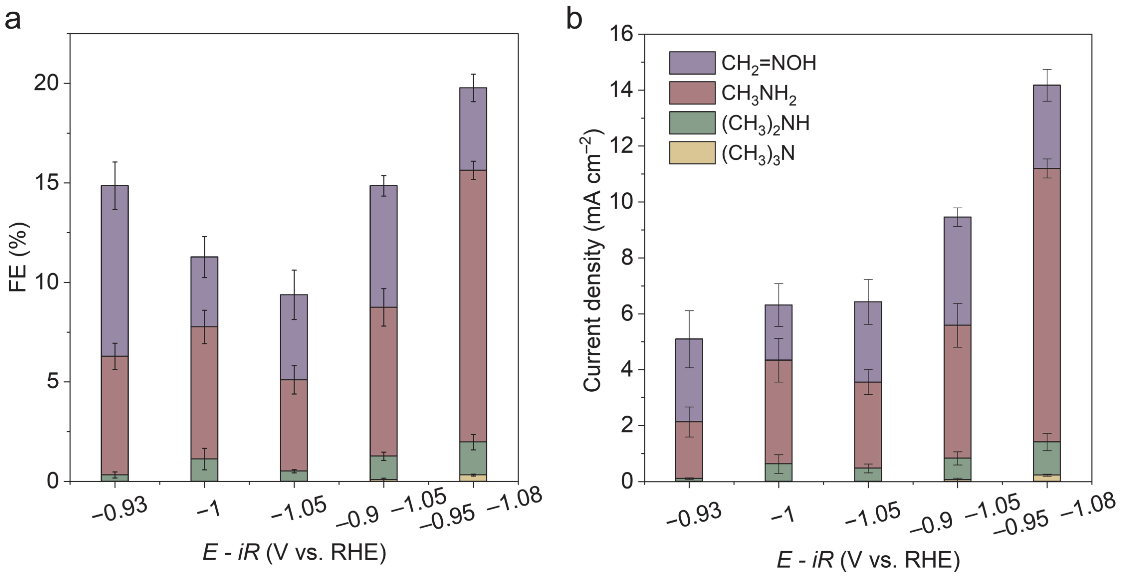

Supplementary Fig. 15 | Faradaic efficiencies (a) and partial current densities (b) of C–N products under static versus optimal pulsed electrolysis conditions. Source data for Supplementary Figure 15 are provided as a Source Data file.

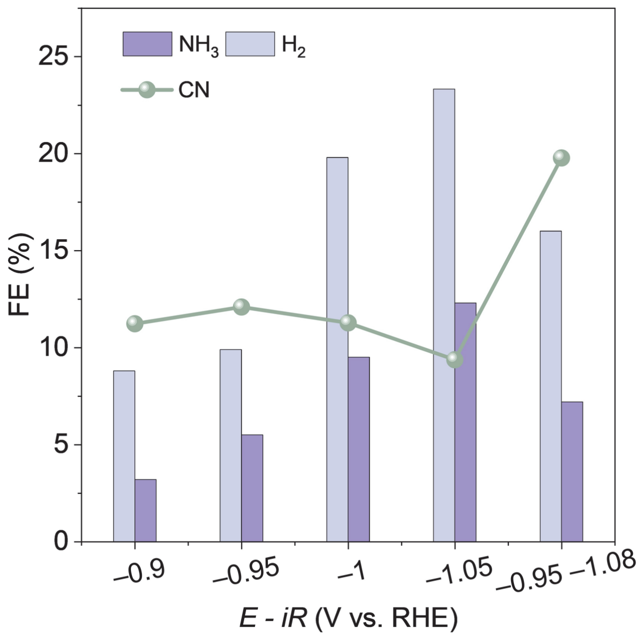

Supplementary Fig. 16 | Faradaic efficiencies of H_2, NH_3 and C–N products for static and pulsed potentials. Source data for Supplementary Figure 16 are provided as a Source Data file.

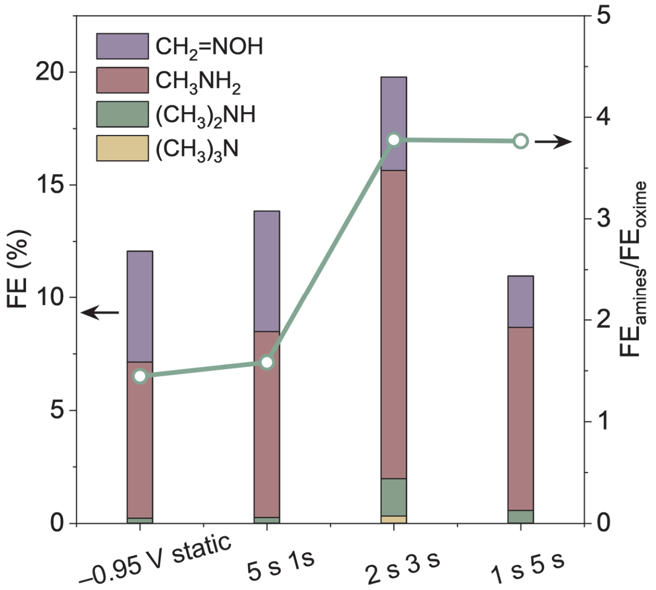

Supplementary Fig. 17 | Faradaic efficiencies of C–N products at static and pulsed potentials with different pulse durations. Source data for Supplementary Figure 17 are provided as a Source Data file.

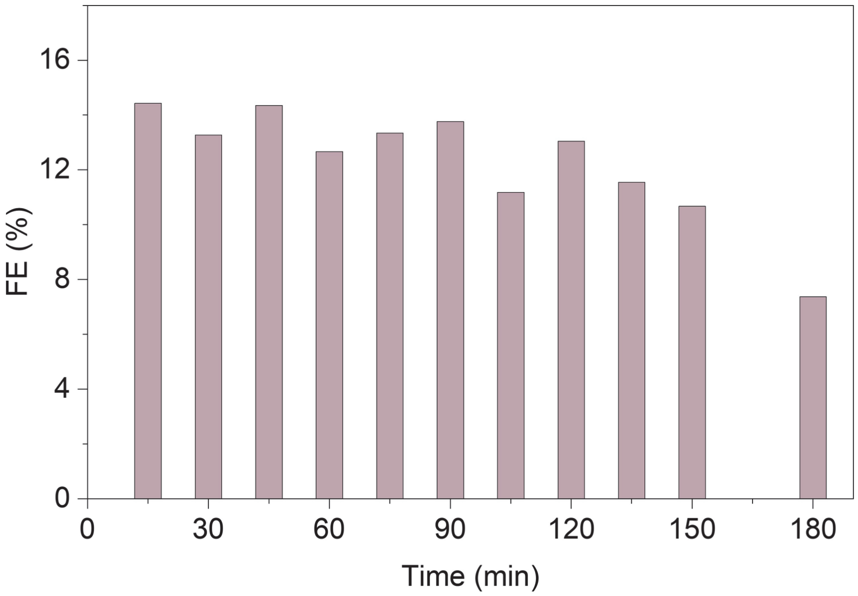

Supplementary Fig. 18 | Faradaic efficiencies of methylamine over time using pulsed electrolysis. Source data for Supplementary Figure 18 are provided as a Source Data file.

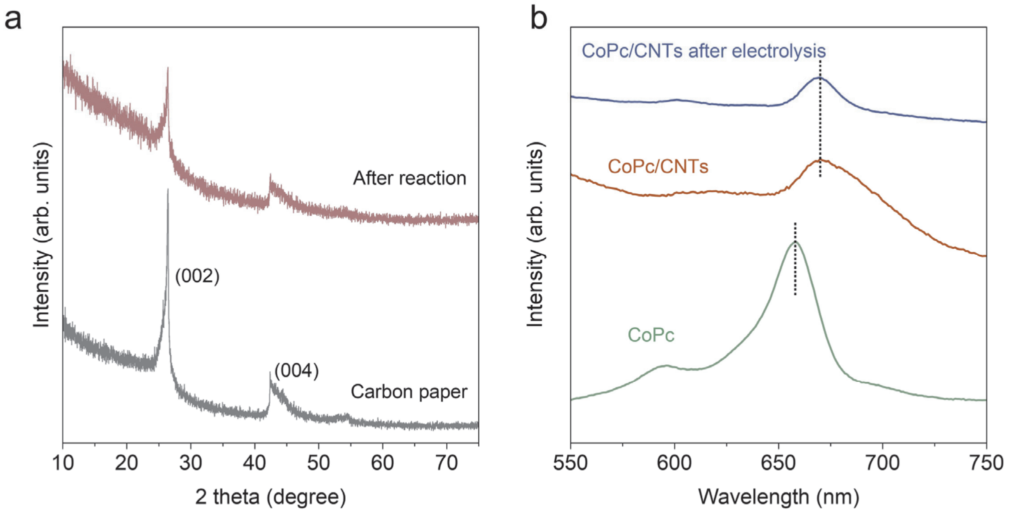

Supplementary Fig. 19 | XRD (a) and UV-Vis (b) of CoPc/CNTs after electrolysis. Source data for Supplementary Figure 19 are provided as a Source Data file.

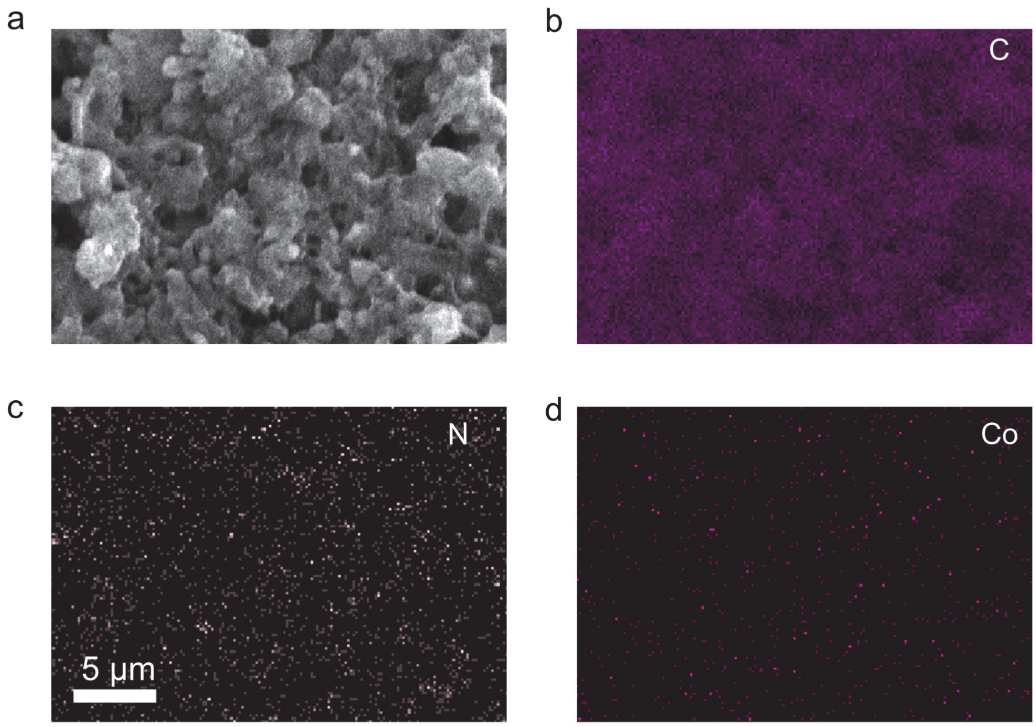

Supplementary Fig. 20 | (a) SEM image of the CoPc/CNTs on carbon paper after electrolysis. (b–d) Corresponding EDS mapping showing the distribution of C, N, and Co.

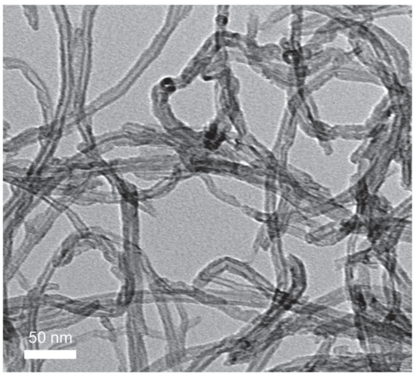

Supplementary Fig. 21 | TEM image of CoPc/CNTs on carbon paper after electrolysis.

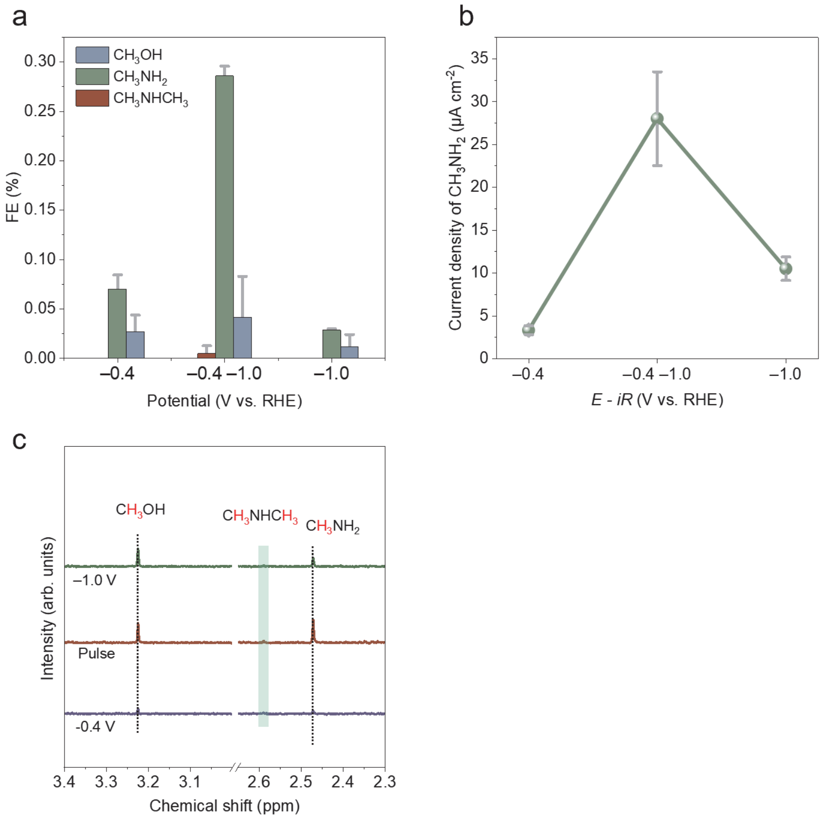

Supplementary Fig. 22 | Faradaic efficiencies of methylamine, dimethylamine, and methanol, and partial current density of methylamine at different applied potentials using Cu catalyst (a, b). The Cu catalyst was prepared by sputter-coating (SPI Supplies, Model 14430) on carbon cloth (MPL-WIS1011, Full Cell Store) at a deposition current of 40 mA for 360 s. Electrolysis was carried out in a home-made gas diffusion electrode cell using 0.1 M $KHCO_3$ and 0.05 M KNO_3 as electrolytes. Corresponding ^{1}H-NMR spectra (c). Error bars represent the standard deviation from at least three independent measurements. Source data for Supplementary Figure 22 are provided as a Source Data file.

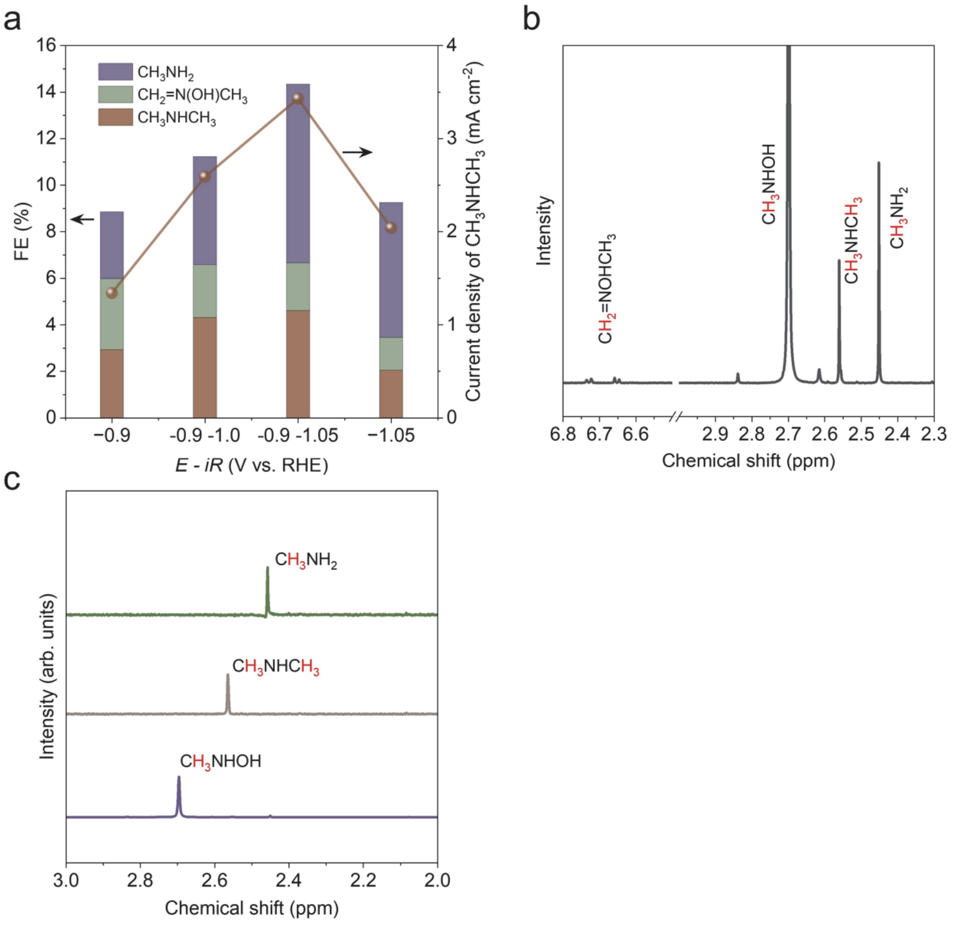

Supplementary Fig. 23 | Faradaic efficiencies of C–N products from CO_2-saturated 0.5 M phosphate-buffered saline (PBS, pH 7) electrolytes containing 50 mM CH_3NO_2, and the partial current density of dimethylamine at different applied potentials using a Cu catalyst (a). ^{1}H NMR spectra of products obtained under pulsed electrolysis conditions (b). ^{1}H NMR spectra of standard methylamine, dimethylamine, and methylhydroxylamine in 0.5 M PBS (c). Source data for Supplementary Figure 23 are provided as a Source Data file.

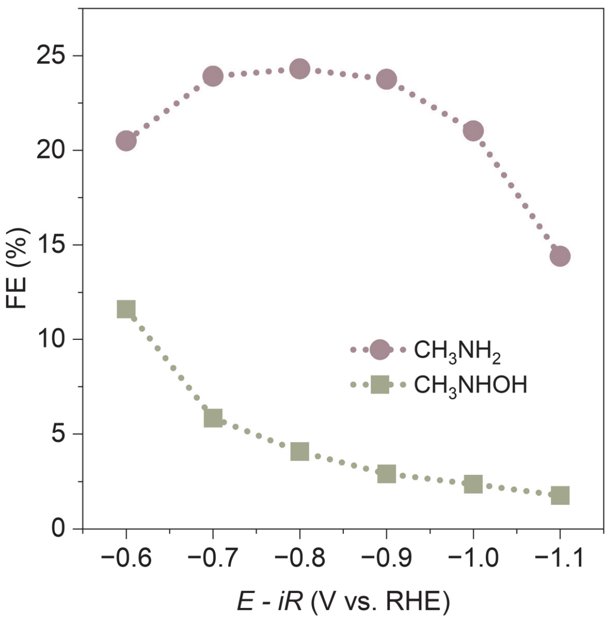

Supplementary Fig. 24 | Faradaic efficiencies of methylamine and N-methylhydroxylamine at different potentials. Source data for Supplementary Figure 24 are provided as a Source Data file.

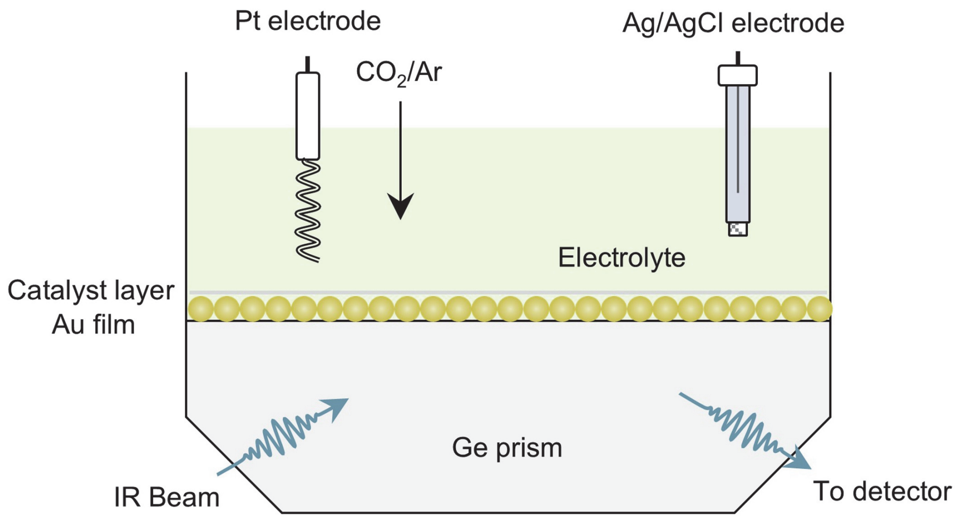

Supplementary Fig. 25 | Schematic of in-situ ATR-SEIRAS measurement setup.

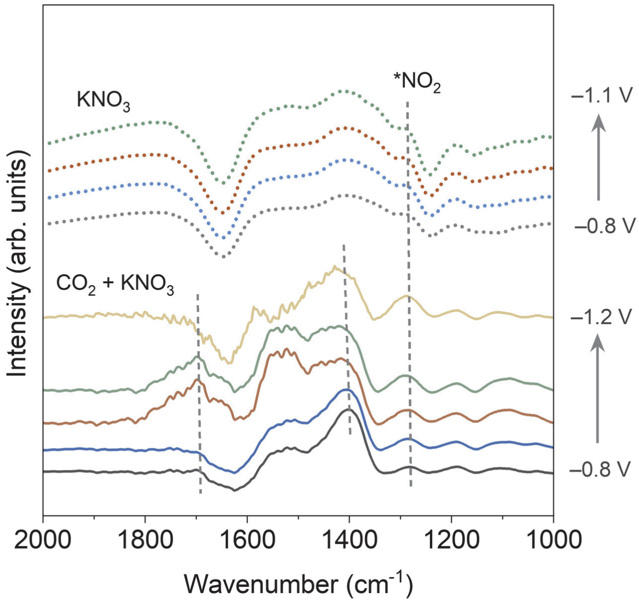

Supplementary Fig. 26 | In-situ ATR-SEIRAS spectra at different potentials: Ar-saturated KNO_3 (dashed line) and CO_2-saturated $KHCO_3$ and KNO_3 (solid line). Although NO_3 adsorption slightly overlaps with the C–N signal, the enhanced peaks under CO_2 indicate C–N coupling. Source data for Supplementary Figure 26 are provided as a Source Data file.

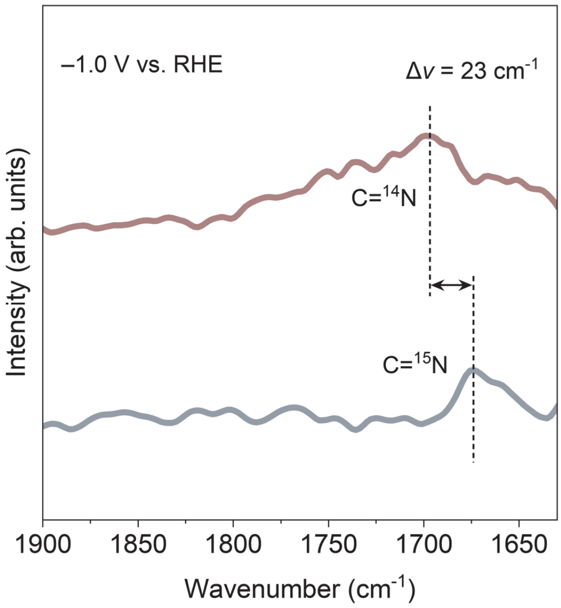

Supplementary Fig. 27 | Comparison of IR spectra at −1.0 V using CO_2-saturated $KHCO_3$ with KNO_3 in H_2O (red line) and $K^{15}NO_3$ in D_2O (green line). The C=^{15}N stretching vibration is red-shifted relative to C=^{14}N due to the isotopic effect, and D_2O was used to avoid overlap with the strong H_2O absorption peak, which would otherwise obscure the C=^{15}N signal. Source data for Supplementary Figure 27 are provided as a Source Data file.

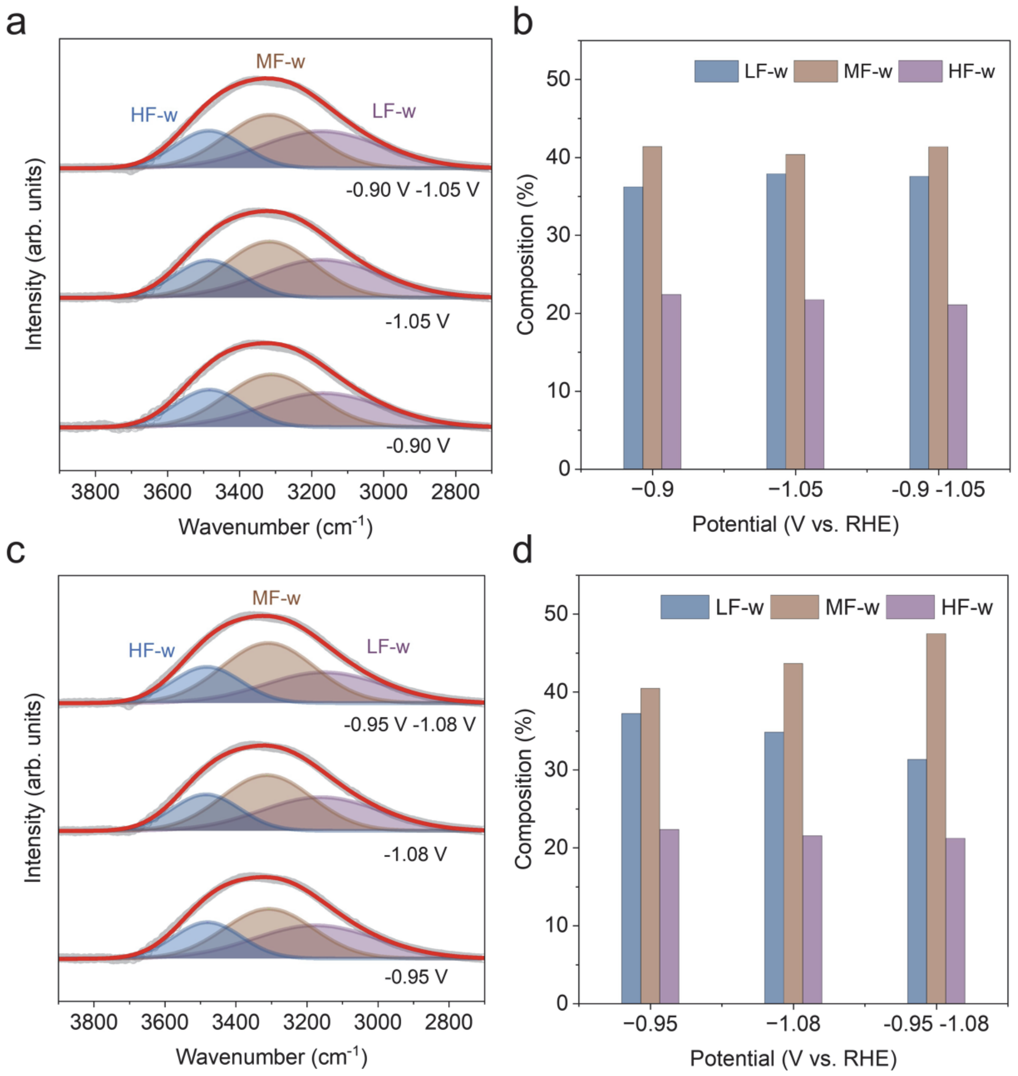

Supplementary Fig 28 | a, c) Spectral composition of interfacial water as a function of potential. The O–H stretching band is deconvoluted into three components: low-frequency water (LF-w), medium-frequency water (MF-w) and high-frequency water (HF-w), which are assigned to fully hydrogen-bonded water, partially hydrogen-bonded water and cation coordinated water, respectively. b, d) Interfacial water compositions under different potentials. Source data for Supplementary Figure 28 are provided as a Source Data file.

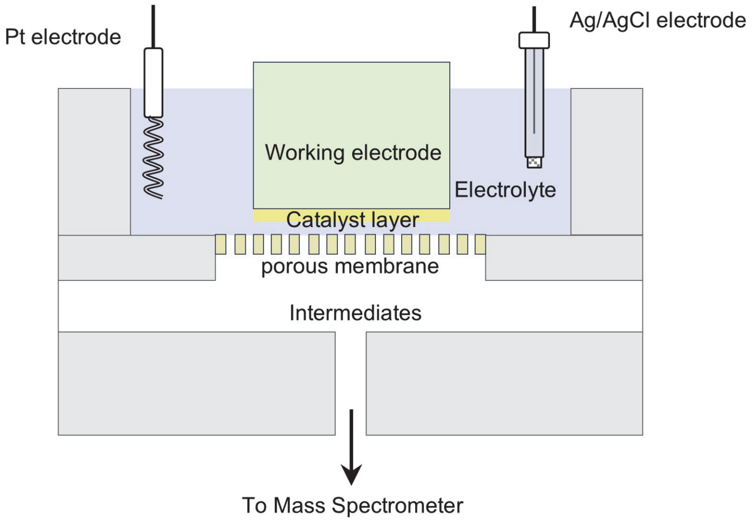

Supplementary Fig. 29 | Schematic of *in-situ* ECMS measurement setup.

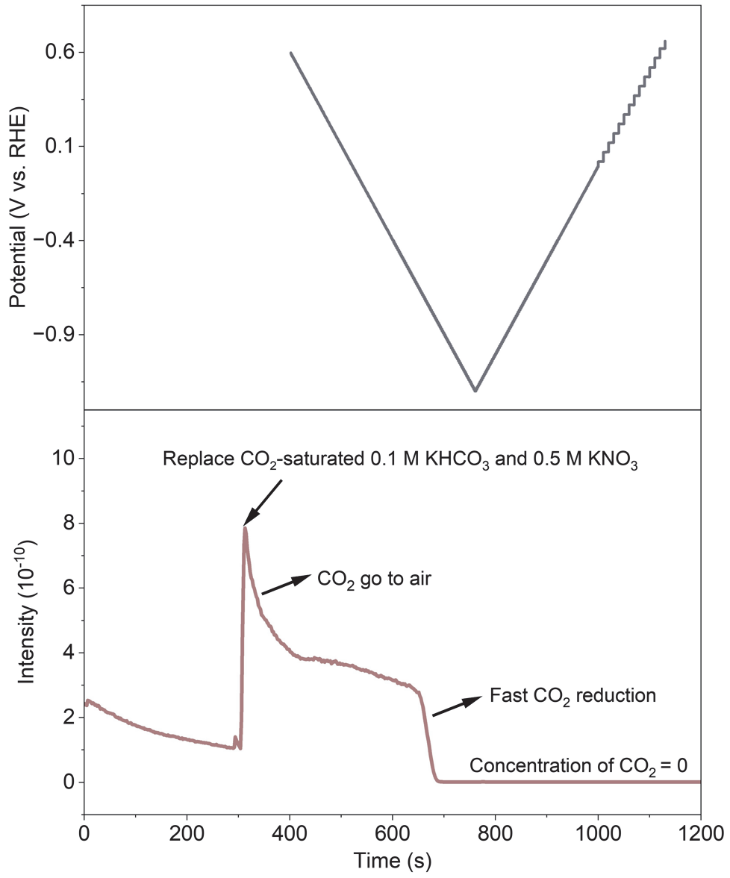

Supplementary Fig. 30 | In-situ ECMS monitoring of CO_2 concentration. Source data for Supplementary Figure 30 are provided as a Source Data file.

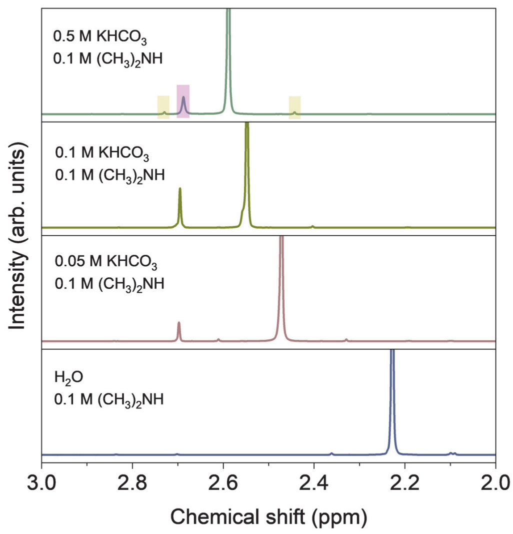

Supplementary Fig. 31 | ^{1}H-NMR of $KHCO_3$ at different concentrations (0, 0.05, 0.1 and 0.5 M) and 0.1 M dimethylamine. For 0.1 M dimethylamine and 0.5 M $KHCO_3$, in addition to the main CH_3 peak at ~2.59 ppm, two small symmetric peaks (~2.73 ppm and ~2.44 ppm) may arise from weak intermolecular interactions or self-association of dimethylamine. A minor peak at ~2.69 ppm in the presence of $KHCO_3$ electrolyte may reflects weak interactions between dimethylamine molecules and ions in solution. Source data for Supplementary Figure 31 are provided as a Source Data file.

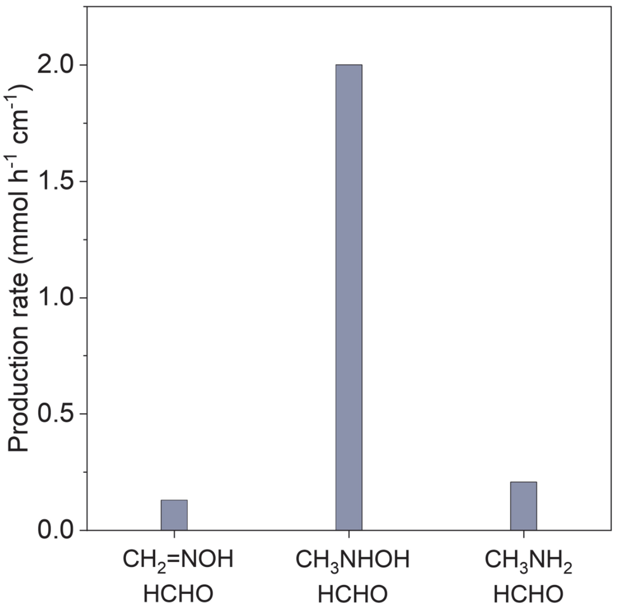

Supplementary Fig. 32 | Production rate of dimethylamine from different N-intermediates (0.1 M) in the presence of 0.1 M HCHO in 0.5 M PBS. Source data for Supplementary Figure 32 are provided as a Source Data file.

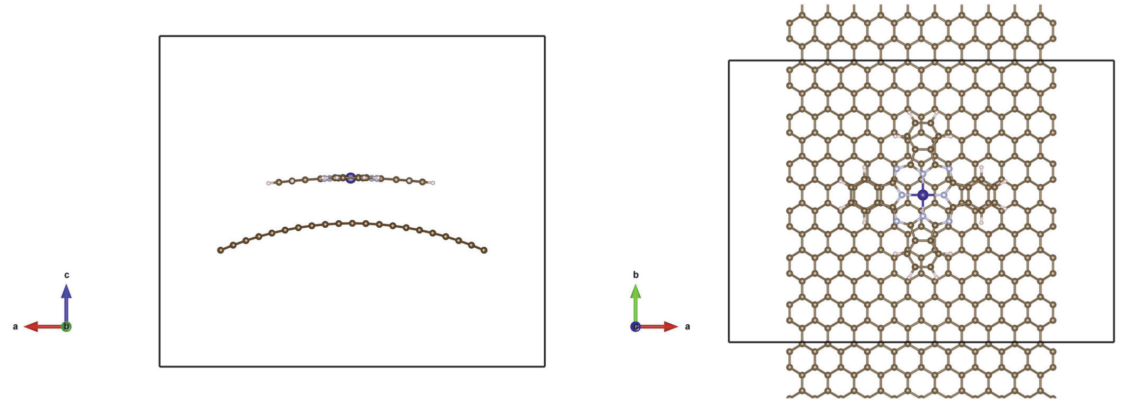

Supplementary Fig. 33 | Side and top views of the optimized CoPc/CNTs.

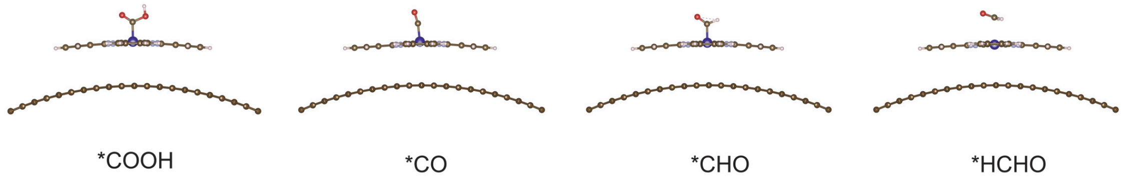

Supplementary Fig. 34 | Configurations of intermediates on CoPc/CNTs during CO_2 reduction to *HCHO.

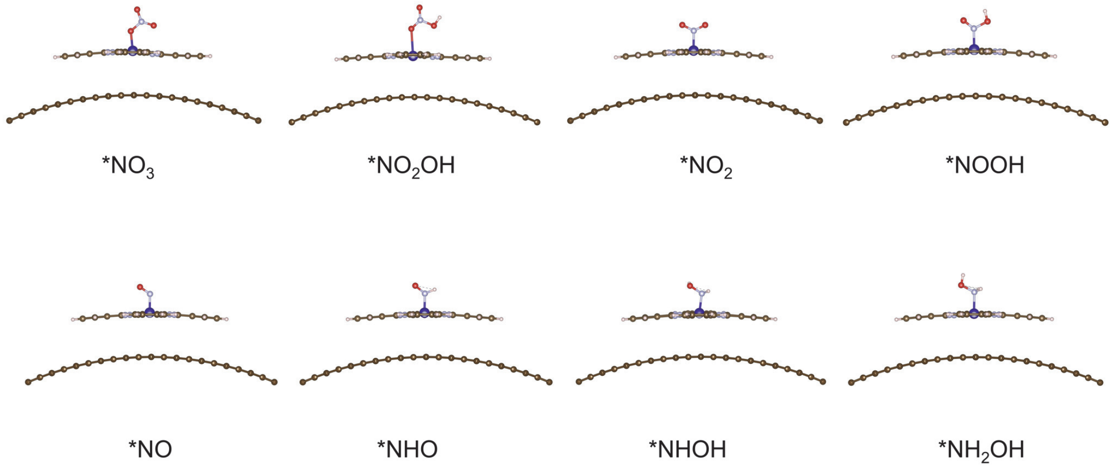

Supplementary Fig. 35 | Configurations of intermediates on CoPc/CNTs during NO_3^- reduction to $*NH_2OH$.

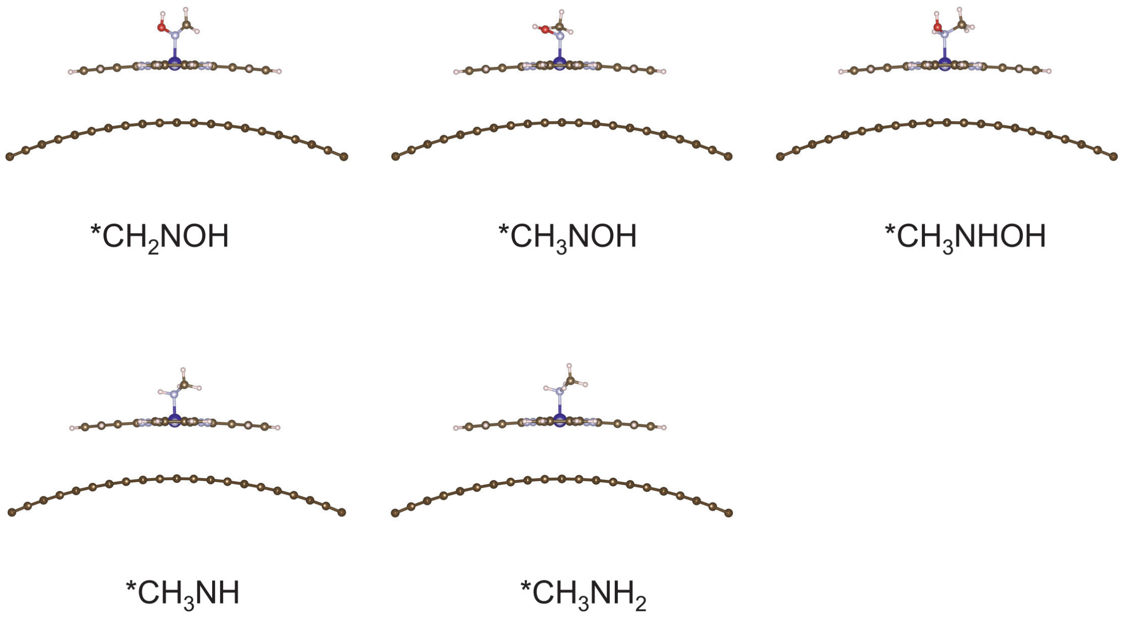

Supplementary Fig. 36 | Configurations of intermediates on CoPc/CNTs during CH_3NH_2 formation from $*HCHO$ and $*NH_2OH$.

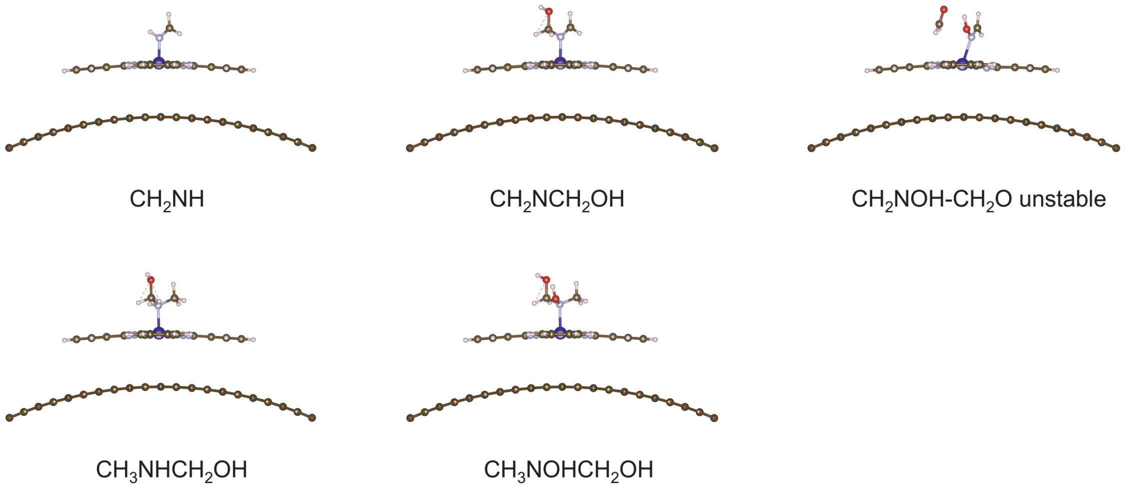

Supplementary Fig. 37 | Configurations of intermediates on CoPc/CNTs during the second C–N formation step.

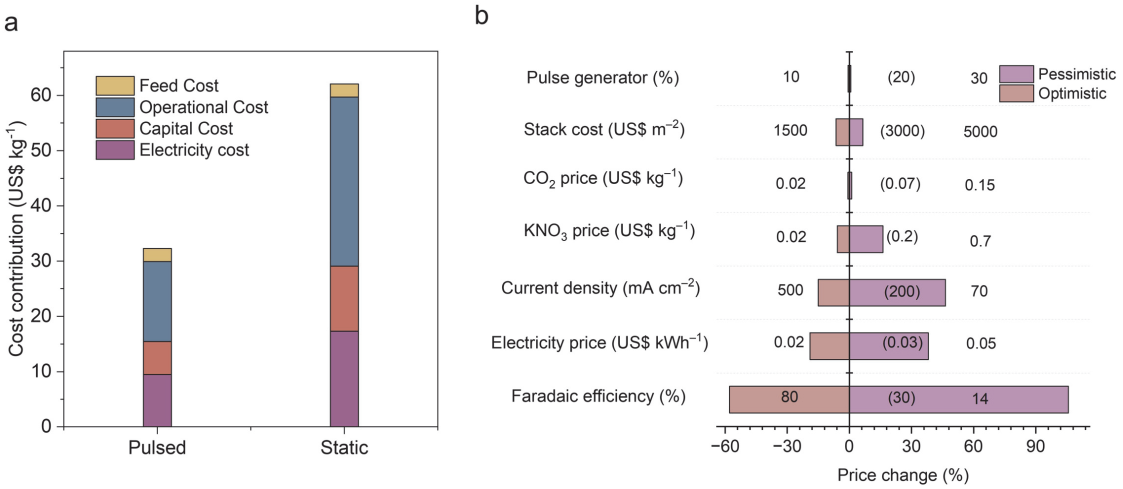

Supplementary Fig. 38 | TEA evaluation of methylamine production. Cost distributions of more reductive pulse and static electrolysis for methylamine production (**a**). Sensitivity analysis for the production cost of methylamine (**b**). The values shown represent the optimistic, base, and pessimistic cases for each parameter. Source data for Supplementary Figure 38 are provided as a Source Data file.

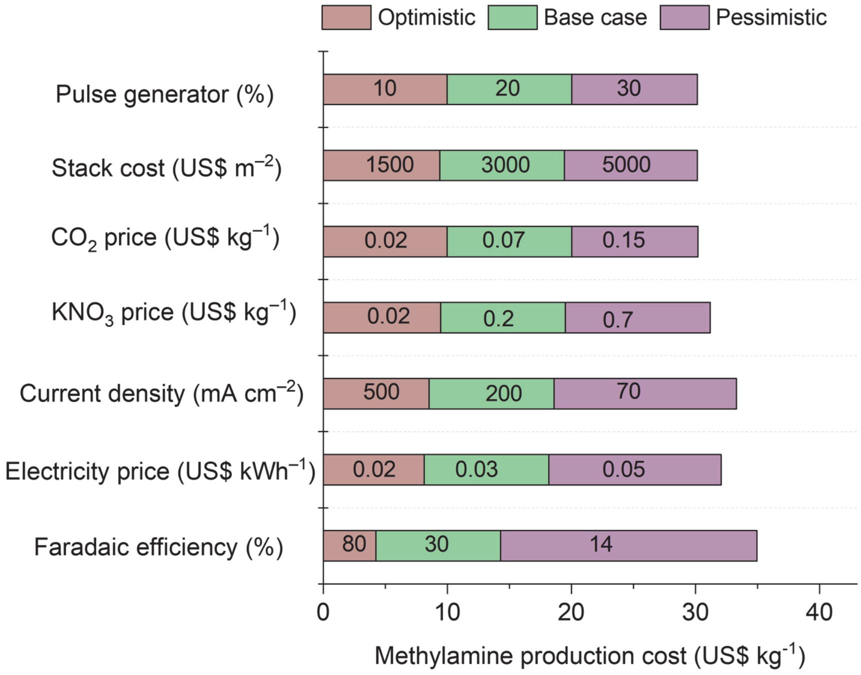

Supplementary Fig. 39 | Sensitivity analysis of the production cost of methylamine. The values shown represent the optimistic, base, and pessimistic cases for each parameter (**Supplemetary Table 5**). Source data for Supplementary Figure 39 are provided as a Source Data file.

Supplementary Tables

Supplementary Table 1 ICP-OES analysis of electrolyte before and after electrocatalysis.

	Potentials	Electrolytes	Concentration of Co (μg/mL)
Before electrolysis	–0.95 V (2s)<br>–1.68 V (3s)	0.5 M KNO_3 + 0.1 M $KHCO_3$	0
After electrolysis	–0.95 V (2s)<br>–1.68 V (3s)	0.5 M KNO_3 + 0.1 M $KHCO_3$	0

Supplementary Table 2 Possible band assignments of *in-situ* ATR-SEIRAS.

Wavenumber (cm^{-1})	Assignment	Intermediate	References
~1285	N−O vibration	$^*NO_2$	Chem Catal., 2023, 3, 100595;[8]<br>Nat. Catal., 2023, 6, 402-414.[9]
~1410	C−N vibration	*C−N	Nat. Chem., 2020, 12, 717–724;[10]<br>ACS Energy Lett., 2023, 8, 3373–3380.[11]
~1630	O–H vibration	H_2O	Adv. Mater., 2024, 36, 2403958.[12]
~1690	C=N vibration	*C=N	J. Am. Chem. Soc., 2024, 146, 19572−19579.[13]

Supplementary Table 3 Possible peak assignments of *in-situ* ECMS.

m/*z*	Intermediate	Reaction	References
2	H_2	H_2 evolution	Nat. Catal., 2023, 6, 807–817.[14]
33	NH_2OH	NO_3^- reduction	Nat. Catal., 2023, 6, 402–414;[9] Adv. Funct. Mater., 2024, 2401194.[15]
17	NH_3	NO_3^- reduction	Nat. Catal., 2023, 6, 402–414.[9]
44	CO_2	reactant	Nat. Commun, 2024, 15, 9781.[16]
28	CO	CO_2 reduction	Nat. Catal., 2023, 6, 807–817.[14]
29	CHO	CO_2 reduction	Chem. Sci., 2011, 2, 1902–1909.[17] Langmuir 2008, 24, 4917–4923.[18]

Supplementary Table 4 Parameters and corresponding values used in the TEA

Parameters	Value
Eletricity price	US$0.03 per kWh
Energy efficiency	80 %
Power of plant	10 MW
Membrane cost	US$3000 m^{-2}
Interest rate	8%
Lifetime of plant	20 years
Installation factor	+20 % of CapEx
Separation investment	+10 % of CapEx
Pulse generator	+20 % of CapEx
Membrane replacement	US$1000 m^{-2}
Separation cost	+30% of electricity cost

Supplementary Table 5 Parameters and corresponding values used in the sensitive analysis

	Optimistic	Base	Pessimistic
FE (%)	80	30	14
Total current density (mA cm^{-2})	500	200	70
Pulse generator (%)	10	20	30
Electricity price (US$ kWh^{-1})	0.02	0.03	0.05
CO_2 price (US$ kg^{-1})	0.02	0.07	0.15
KNO_3 price (US$ kg^{-1})	0.02	0.2	0.7
Stack cost (US$ m^{-2})	1500	3000	5000

Supplementary References

1 Jouny, M., Luc, W. & Jiao, F. General techno-economic analysis of CO_2 electrolysis systems. *Ind. Eng. Chem. Res.* **57**, 2165–2177 (2018).

2 Lee, S. *et al.* Techno-economic analysis and life-cycle assessment of the electrochemical conversion process with captured CO_2 in an amine-based solvent. *Green Chem.* **25**, 10398–10414 (2023).

3 *https://www.intratec.us/chemical-markets/potassium-nitrate-price*.

4 Mayyas, A. T., Ruth, M. F., Pivovar, B. S., Bender, G. & Wipke, K. B. Manufacturing cost analysis for proton exchange membrane water electrolyzers. (National Renewable Energy Laboratory (NREL), Golden, CO (United States), 2019).

5 Barecka, M. H., Ager, J. W. & Lapkin, A. A. Techno-economic assessment of emerging CO_2 electrolysis technologies. *STAR Protocols* **2**, 100889 (2021).

6 Spurgeon, J. M. & Kumar, B. A comparative technoeconomic analysis of pathways for commercial electrochemical CO_2 reduction to liquid products. *Energy Environ. Sci.* **11**, 1536–1551 (2018).

7 Shin, H., Hansen, K. U. & Jiao, F. Techno-economic assessment of low-temperature carbon dioxide electrolysis. *Nat. Sustain.* **4**, 911–919 (2021).

8 Guiet, A. *et al.* Reversible transition of an amorphous Cu-Al oxyfluoride into a highly active electrocatalyst for NO_3^- reduction to NH_3. *Chem Catal.* **3**, 100595 (2023).

9 Han, S. *et al.* Ultralow overpotential nitrate reduction to ammonia via a three-step relay mechanism. *Nat. Catal.* **6**, 402–414 (2023).

10 Chen, C. *et al.* Coupling N_2 and CO_2 in H_2O to synthesize urea under ambient conditions. *Nat. Chem.* **12**, 717–724 (2020).

11 Wang, Y. *et al.* Spatial management of CO diffusion on tandem electrode promotes NH_2 intermediate formation for efficient urea electrosynthesis. *ACS Energy Lett.* **8**, 3373–3380 (2023).

12 Qi, S. *et al.* Ultrathin high-entropy Fe-based spinel oxide nanosheets with metalloid band structures for efficient nitrate reduction toward ammonia. *Adv. Mater.* **36**, 2403958 (2024).

13 Pan, Y. *et al.* Electrocatalytic coupling of nitrate and formaldehyde for hexamethylenetetramine synthesis via C–N bond construction and ring formation. *J. Am. Chem. Soc.* **146**, 19572–19579 (2024).

14 Zhang, H., Gao, J., Raciti, D. & Hall, A. S. Promoting Cu-catalysed CO_2 electroreduction to multicarbon products by tuning the activity of H_2O. *Nat. Catal.* **6**, 807–817 (2023).

15 Su, J. *et al.* Engineering the metal-support interaction and oxygen vacancies on Ru@P-Fe/Fe_3O_4 nanorods for synergetic enhanced electrocatalytic nitrate-to-ammonia conversion. *Adv. Funct. Mater.* **34**, 2401194 (2024).

16 Ye, K. *et al.* Molecular level insights on the pulsed electrochemical CO_2 reduction. *Nat Commun* **15**, 9781 (2024).

17 Schouten, K., Kwon, Y., Van Der Ham, C., Qin, Z. & Koper, M. A new mechanism for the selectivity to C_1 and C_2 species in the electrochemical reduction of carbon dioxide on copper electrodes. *Chem. Sci.* **2**, 1902–1909 (2011).

18 Na, K. *et al.* “Smart” biopolymer for a reversible stimuli-responsive platform in cell-based biochips. *Langmuir* **24**, 4917-4923 (2008).
