# Supplementary material for: Pulsed electrosynthesis orthogonally optimizes C‒N coupling and hydrogenation for amine production with a molecular catalyst
Source: Nat Commun. 2026 May 4;17:4027. doi: 10.1038/s41467-026-72678-0 (PMC13139404; doi:10.1038/s41467-026-72678-0)
Supplement: Supplementary file 2 — Description of Additional Supplementary Files [file 41467_2026_72678_MOESM2_ESM.pdf]

Description of Additional Supplementary Files

File Name: Supplementary Data 1

Description: Atomic coordinates of calculation models.
